# Environmental Risk in American Indian Children, Including Cardiovascular and Hematologic Consequences of Cadmium Exposure: Possible Means of Mitigation

**DOI:** 10.3390/ijerph22091437

**Published:** 2025-09-16

**Authors:** Joseph Burns, Cesar E. Larancuent, Cian L. Jacob, Danielle A. Heims-Waldron, Whitney R. Lloyd, Justin P. Zachariah, Abraham Haimed, Ana Navas-Acien, Jason F. Deen

**Affiliations:** 1 Division of Pediatric Cardiology, Texas Children’s Hospital, Baylor College of Medicine, Houston, TX 77030, USA; justin.zachariah@bcm.edu; 2 Divisions of Medicine and Pediatrics, Texas Children’s Hospital, Baylor College of Medicine, Houston, TX 77030, USA; cesar.larancuent@bcm.edu; 3 Herbert Wertheim College of Medicine, Florida International University, Miami, FL 33199, USA; cjaco066@med.fiu.edu; 4 Division of Pediatrics, Boston Children’s Hospital, Boston, MA 02115, USA; danielle.heims-waldron@childrens.harvard.edu; 5 Division of Pediatric Cardiology, Stanford University, Paolo Alto, CA 94304, USA; lloyd191@stanford.edu; 6 Division of Pediatric Hematology/Oncology, Children’s Hospital of Philadelphia, Philadelphia, PA 19104, USA; haimeda@chop.edu; 7 Department of Environmental Health Sciences, Columbia University Mailman School of Public Health, New York, NY 10032, USA; an2737@cumc.columbia.edu; 8 Divisions of Pediatric and Adult Cardiology, Seattle Children’s Hospital, University of Washington, Seattle, WA 98105, USA; jason.deen@seattlechildrens.org

**Keywords:** environmental health, pediatric cardiology, pediatric hematology, American Indian/Alaska Native, health inequity

## Abstract

Emerging evidence reveals that cadmium exposure is associated with hypertension, cardiovascular disease (CVD), and anemia, among others. Toxic metal exposure poses a particular threat to American Indian/Alaska Native populations, particularly given their proximity to mining waste. This review aims to summarize and organize evidence explaining the cardiovascular and hematologic consequences of cadmium exposure in children, including specific data on this exposure in AI/AN people, and strategies and policy actions to mitigate these consequences in AI/AN children.

## 1. Introduction

### 1.1. Toxic Metal Exposure as a Social Driver of Health

Communities exposed to environmental hazards are at a heightened risk of adverse health outcomes [1]. In an umbrella review of 1353 articles, 103 studies identified associations between 69 environmental risk factors and health conditions [1]. These factors were categorized into air pollution, residential exposures, chemicals and contaminant metals, physical exposures, and tobacco smoke [1]. A meta-analysis identified significant links between these environmental exposures and numerous health outcomes, including asthma, low birth weight, and cardiovascular mortality [1].

Multiple reviews have described the cardiovascular impacts of contaminant metal exposure, underscoring associations between heavy metal exposure and hypertension, atherosclerosis, arrhythmia, or increased cardiovascular disease event risk [2,3]. The metals implicated included cadmium, lead, nickel, chromium, arsenic, selenium, zinc, copper, barium, and mercury [2]. For most of these heavy metals, ingestion of food and water contaminated by industrial sources is the primary exposure route [2,4].

The adverse health effects of environmental exposures disproportionately affect disadvantaged populations. Studies reveal that low-income communities and racial/ethnic minority groups are more exposed to social inequality, poverty, and poor housing quality [5]. This implies that these communities are less well-equipped to remediate climate change-related hazards despite an increased burden of exposure due to proximity to toxic waste.

Numerous studies have highlighted the inequitable distribution of environmental hazards, including hazardous waste sites, industrial facilities, and sewage treatment plants, in socially disadvantaged neighborhoods [5]. The differential exposure in disadvantaged populations illustrates how environmental justice drives health outcomes.

### 1.2. Introduction to American Indian Health Inequity

American Indian/Alaska Native (AI/AN) people have worse health outcomes than the general population [6]. This extends to children, where health inequities have been described in physical, mental, and social domains [7]. AI/AN children have among the highest rates of obesity, cardiovascular disease (CVD) risk factors, and diabetes mellitus relative to other American children [7]. These inequities are inextricably tied to unique adverse social drivers of health affecting AI/AN communities, including settler colonialism, which has driven land allocation patterns in the United States [8].

Settler colonialism describes the process by which newcomer populations dislodge existing populations through destabilization of people and cultures [8]. In the United States, for example, this principle has been instantiated through numerous specific actions, including forced relocations of multiple tribes that were materially and culturally adapted to indigenous and then newcomer-recognized resource-rich ancestral homelands to resource-poor regions [8]. Further degradation through waste disposal or high-pollution industries is often directed to these already economically devalued areas, where the perceived additional harm is minimal. This disruption of relationships with the land is further compounded by the complexities of tribal sovereignty, which is also subject to state and federal legislation, limiting the capacity of tribal governments to self-govern and mitigate degradation [8].

### 1.3. Cadmium Sources and Exposures

Cadmium is a toxic heavy metal that is naturally present in lead and zinc ores, and arises from cigarette smoke, contaminated food and water, and pollution from smelting industries [2]. Diet is the primary source of cadmium intake for most individuals [9]. In a 2018 cross-sectional study using National Health and Nutrition Examination Survey (NHANES) data from 2007 to 2012, researchers found that cereals and bread were the top contributors to daily cadmium intake among Americans aged 2 years and older [9]. In addition, dietary cadmium sources varied by sociodemographic group [9]. For example, young children were more likely to consume cadmium through foods such as peanuts, strawberries, cookies, and milk compared to older adults [9]. Recent data also suggests rice, especially infant rice cereals used as the introduction to solid food, may be strongly contaminated with cadmium [10].

Cadmium is also commonly found in mining waste, often negligently left at sites of previous mines for heavy and radioactive metals. Cadmium may then enter water supplies through direct infiltration or runoff due to flooding and erosion—a critical consideration in the context of ongoing global climate change [11]. A 2024 study evaluated gold mining waste across 30 different sites in the Victorian Goldfields, Australia, and the investigators noted that the cadmium concentrations were 2.4 times higher than the reference value [12]. A 2018 study identified elevated cadmium blood concentrations of 7.7 μg/L in children living near a lead–zinc mine in Zambia [13]. A similar 2020 study investigated the cadmium levels near a lead–zinc mine and copper smelter in Jiangxi Province, China [14]. The investigators calculated that the mean hair and urine cadmium concentrations were “0.0.098 ± 0.10 mg kg^−1^ and 5.7 ± 3.1 μg L^−1^ in the mining area, and 0.30 ± 0.21 mg kg^−1^ and 5.5 ± 3.5 μg L^−1^ in the smelting area, respectively” [14].

A 2019 study further emphasized pediatric cadmium exposure, reporting estimated mean intakes ranging from 0.38 to 0.44 µg/kg body weight per day [15]. Another evaluation reported that pediatric cadmium levels have remained steady from 2004 to 2008 [15]. These values, however, exceed the European Food Safety Authority’s tolerable weekly intake threshold of 2.5 µg/kg/week (approximately 0.36 µg/kg/day) [16]. The United States Department of Agriculture does not define a safe cadmium intake threshold.

Cadmium is known to generate reactive oxygen species by the depletion of glutathione, which can affect DNA repair and directly inhibit oxidative phosphorylation at the level of the mitochondria [17]. It additionally inhibits antioxidant enzymes, impairs cellular calcium levels, and affects caspase and protein kinase enzymes [17]. These broad mechanisms of toxicity result in multisystem illness, including cardiovascular, hematologic, nervous, urinary, reproductive, and respiratory disease [17].

### 1.4. Cadmium Exposure in American Indian/Alaska Native People

AI communities have higher cadmium exposure than the general population [18]. More than 160,000 abandoned hard rock mines are located on AI lands throughout the western United States, a consequence of poorly rehabilitated mines from the mid-1900s, contaminating soil and water in AI communities [18]. Environmental exposures from inhaling airborne particulate matter containing cadmium often result in higher cadmium biochemical profiles in AI people in the area compared to adults of a similar age from urban areas in the United States [19].

Several AI/AN groups have higher rates of tobacco use than any other racial/ethnic subgroup in adults and children in the United States, with smoking being one of the primary sources of cadmium exposure [20]. Food remains one of the primary sources of cadmium exposure. An SHFS evaluation of dietary sources of cadmium, including children ages 15 and older, found a statistically significant association between processed meat consumption and urinary cadmium [21].

Thus, based on land allocation patterns and inequitable exposures, AI/AN people may be inequitably affected by the consequences of complex cadmium exposure, including the leaching of mining waste into soil and water.

## 2. Methods

This review aims to summarize and organize evidence describing the cardiovascular and hematologic consequences of cadmium exposure in children, including specific data on this exposure in AI/AN people, and strategies and policy actions to mitigate these consequences in AI/AN children.

**Figure 1:**
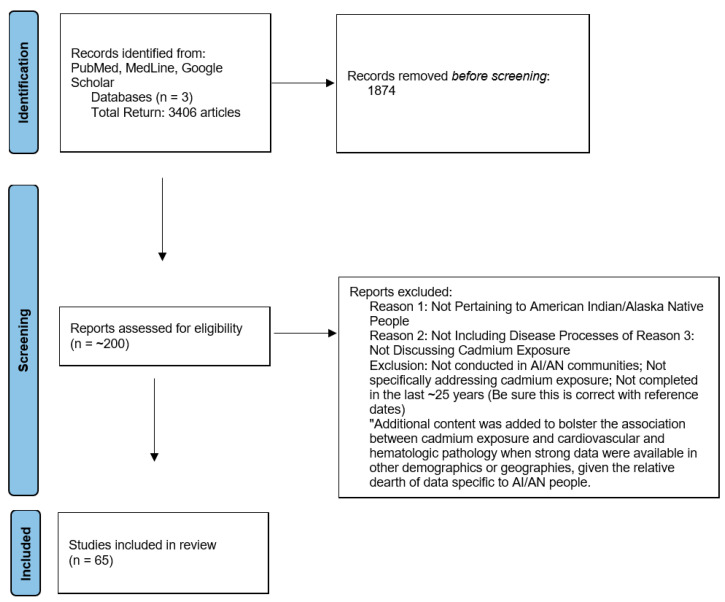
PRISMA diagram summarizing search strategy and yield [22].

All references were derived from broad searches of terms including “cadmium”, “cardiovascular disease”, “congenital heart disease”, “hematologic”, and “American Indian/Alaska Native” in the largest publication databases, including PubMed, MedLine, and Google Scholar, to retrieve the broadest returns given the scoping nature of this review. Specific inclusion criteria required pertinency to AI/AN populations, data explicitly reporting cadmium exposure, not necessarily other heavy metals, and describing effects in cardiovascular and hematologic health. Articles released within the last ten years, with exceptions, were then critically appraised by the authors to determine appropriateness for inclusion in this review, as well as for repeated data. The pertinent articles were then reviewed and summarized in the subsections below. Additional content was added to bolster the association between cadmium exposure and cardiovascular and hematologic pathology when strong data were available in other demographics or geographies, given the relative dearth of data specific to AI/AN people. This strategy is summarized in Figure 1.

## 3. Cardiovascular and Hematologic Consequences of Cadmium Exposure in Childhood

### 3.1. Association of Cadmium with Congenital Heart Disease and Cardiovascular Disease

Cadmium has been associated with the increased incidence of congenital heart disease in the children of exposed mothers [23,24,25,26].

Cadmium is known to cross the placental barrier, thought to be transferred in breast milk, and has been associated with congenital heart disease [27,28,29]. A study found that human placenta from a mother who was a smoker compared to a non-smoker had a 10- to 20-fold increase in cadmium levels compared to the level in maternal blood, suggesting that the fetus has greater exposure in utero relative to the mother [30]. Using maternal hair samples, a dose-dependent relationship was established, with a nearly three-fold increase in the incidence of conotruncal defects in the highest-exposed sample [23,29]. Other evidence suggests that cadmium exposure is also associated with septal defects [24]. Exposure to other metals, including arsenic, confers a synergistic exposure, yielding a nine-fold higher risk of congenital heart disease than non-exposed mothers [28].

In animal models, the offspring of cadmium-exposed mice were found to have an increased heart weight and were more susceptible to hypertension in adulthood [31]. In humans, a study in Mexico found that increased maternal urinary cadmium levels were not associated with childhood or adolescent blood pressure or birth weight [32]. This finding was further substantiated by an NHANES study, which found an inverse relationship between urinary cadmium and blood pressure in children and adolescents [33]. As cadmium displaces calcium and calcium contributes to vascular tone, this may present a physiologic mechanism. However, dysregulated calcium may also contribute to arterial calcification in adulthood, suggesting a predisposition to early-onset hypertension and CVD.

Generally, cadmium exposure is understudied and warrants further investigation into both the epidemiology of exposure and associations with congenital heart disease and cardiovascular disease.

### 3.2. Cadmium and Pediatric Hematologic Pathology

Cadmium exposure in children has been associated with several hematologic abnormalities, including immune dysregulation, anemia, and potential leukemogenic effects [34,35,36,37,38]. Experimental studies demonstrate that cadmium disrupts hematopoiesis by impairing the function of hematopoietic stem cells and altering the bone marrow microenvironment, leading to increased myelopoiesis at the expense of lymphocyte production [34]. Such changes contribute to weakened immune responses in mouse models [35].

Cadmium also appears to interfere with erythropoiesis through multiple mechanisms. Animal studies report hemolysis, oxidative damage to erythrocyte membranes, and suppression of erythropoietin production [36,37,38]. Reactive oxygen species may also affect the health of the vasculature. Together, these effects contribute to the development of anemia in populations chronically exposed to cadmium.

Epidemiologic data linking cadmium to childhood leukemia are limited but suggestive. A Spanish case–control study found an association between higher cadmium levels in residential topsoil and an increased incidence of pediatric leukemia [39].

Given cadmium’s long biological half-life, ranging from 10 to 30 years, its accumulation in the human body is gradual and persistent, with primary exposure sources including diet and tobacco smoke [40,41]. While accumulation occurs more slowly in children, their relatively higher cadmium intake per body weight and ongoing hematopoietic development may confer disproportionate risk [42].

Emerging evidence suggests that cadmium disrupts hematologic development in children, warranting further investigation into its role in pediatric disease.

### 3.3. Other Health Consequences of Cadmium Exposure in Childhood

Cadmium exposure in childhood can also lead to other health consequences, such as neurocognitive impairment, bone concerns, and impaired kidney function. A 2012 cohort study in rural Bangladesh investigated cadmium exposure in preschool children, and they determined that increased cadmium exposure during pregnancy was inversely correlated with neurodevelopmental outcomes at 5 years [43]. These outcomes included verbal IQ, performance IQ, and full-scale IQ [43]. Concurrent childhood exposure was also associated with behavior difficulties, which were assessed by the Strengths and Difficulties Questionnaire [43].

Bone toxicity is one of the hallmarks of cadmium’s health effects, although most studies have been conducted in adulthood [4]. A 2019 longitudinal study investigated how cadmium exposure in early life affects bone health [44]. They assessed cadmium exposure in urine across a 504 mother–child cohort in Bangladesh and concluded that cadmium exposure was associated with altered bone biomarkers that enhance bone resorption, decreased vitamin D levels, and reduced growth velocity [44].

A 2020 study also investigated the impact of cadmium exposure in childhood on glomerular kidney function. The investigators recruited 601 children from the Programming Research in Obesity, Growth, Environment, and Social Stressors (PROGRESS) cohort in Mexico and determined that high dietary cadmium levels were inversely correlated with blood urea nitrogen levels [45]. Additionally, the investigators asserted that when high levels of cadmium accumulate, more cadmium becomes unbound to metallothionein. This, in turn, can lead to kidney tubular damage [45].

### 3.4. Cardiovascular and Hematologic Consequences of Cadmium Exposure Across the Lifespan

Chronic exposure to cadmium has also been associated with cardiovascular and hematologic disease in adulthood.

The correlation of cadmium with death from hypertension and CVD was first described in 1966 [46]. It has been broadly associated with hypertension, atherosclerosis, and systolic dysfunction [47]. In adulthood, cadmium exposure, measured in blood and urine, has been associated with an increased risk of cardiovascular mortality [48]. Utilizing NHANES, a study evaluating cardiovascular-related biomarkers and cadmium exposure found that higher urinary cadmium was associated with higher C-reactive protein, low-density lipoprotein, non-high-density lipoprotein, and gamma-glutamyltransferase [49]. Another NHANES study found that increasing urine cadmium levels were positively associated with mortality from CVD, heart disease, and coronary artery disease [50]. A study of a Flemish population also supports the relationship between cadmium exposure and left ventricular systolic dysfunction, but notes that the effect size was small and may be affected by lead exposure, highlighting a complication of observational environmental epidemiology in identifying specific associations in mixed exposures and thereby risk implicating none [51]. Pooled data from twelve studies support this association, with increased cadmium exposure linked to excess relative risks of 36% for cardiovascular disease and 30% for coronary artery disease [52].

Chronic cadmium exposure has well-documented hematologic consequences, particularly the development of anemia, which emerges through multiple, compounding mechanisms. These include hemolysis, dysregulation of iron metabolism, and suppression of erythropoietin (EPO) production secondary to renal tubular injury [36,53]. In animal models, prolonged cadmium administration induces hemolytic anemia, tissue iron accumulation, and a progressive decline in EPO synthesis as renal damage worsens—findings that mirror clinical observations in individuals with chronic cadmium intoxication, such as those affected by itai-itai disease, a historical outbreak of cadmium toxicity manifesting as osteomalacia and renal dysfunction [36,53]. Cadmium also accelerates the clearance of senescent erythrocytes, increases oxidative stress within red blood cells, and impairs erythropoiesis in the bone marrow, particularly at higher exposure levels [37,54]. Beyond these effects, cadmium disrupts hematopoietic stem and progenitor cell (HSPC) populations, contributing to long-term immune dysregulation and increased susceptibility to hematologic malignancies over time [26].

Over a lifetime, this cumulative exposure may lead to sustained anemia, impaired erythropoiesis, and altered immune competence, particularly in vulnerable populations [36,41,54,55].

## 4. American Indian/Alaska Native Cadmium Exposure and Consequences

### 4.1. Cardiovascular Health and Cadmium Exposure in AI/AN People

The most robust evidence describing cadmium exposure and cardiovascular health is generated from the Strong Heart Family Study (SHFS), a large longitudinal cohort study of AI people in Arizona, Oklahoma, North Dakota, and South Dakota [52].

An SHFS evaluation found that participants had higher urinary cadmium levels than the general population [21]. Using urinary cadmium, comparing the twentieth to the eightieth percentile of concentrations yielded a hazard ratio of 1.43 for cardiovascular mortality and 1.34 for coronary artery disease mortality [52]. This study also revealed hazard ratios for CVD, coronary artery disease, and heart failure of 1.24, 1.22, and 1.39, respectively [52]. As previously discussed, this increased exposure to cadmium leads to an increase in the incidence of conotruncal defects and congenital heart defects in general in children who have been exposed in utero [23,28,29,30].

Another SHFS project found that higher urinary cadmium levels were associated with elevated systolic and diastolic blood pressure, recognizing this population may have a different risk profile due to other metal exposures and social drivers [56].

The key studies describing cadmium exposure and cardiovascular health in AI/AN people are summarized in Table 1.

### 4.2. Hematologic Health and Cadmium Exposure in AI/AN People

Although the hematologic effects of cadmium exposure have been well described in pediatric and experimental models, direct evidence linking cadmium to hematologic disease in AI/AN populations remains limited. Nonetheless, AI/AN communities experience inequitable cadmium exposure, with data from the Strong Heart Study showing elevated blood and urinary cadmium levels across multiple tribal populations, even among people who have never smoked, suggesting environmental and dietary sources beyond tobacco [21,56,59]. This elevated exposure, combined with known hematologic effects of cadmium in experimental and pediatric populations, raises concern for underrecognized impacts in AI/AN children, specifically, where ongoing hematopoietic development may amplify risk.

To date, no studies have documented a direct relationship between cadmium and hematologic malignancies or primary hematologic disorders in AI/AN populations. However, cadmium’s well-established nephrotoxicity may contribute to indirect hematologic effects [36,53,60]. Chronic kidney damage from cadmium can suppress erythropoietin production and disrupt iron homeostasis, both of which contribute to anemia in exposed populations [36,37,38]. While AI/AN-specific pediatric data are lacking, these mechanisms, particularly those mediated through renal impairment, may carry heightened implications for children due to their ongoing hematopoietic development.

More broadly, cadmium exposure in AI/AN communities has been associated with accelerated epigenetic aging and increased cancer risk, including lung and pancreatic malignancies, particularly among individuals with diabetes [61,62].

These findings underscore the systemic consequences of cadmium exposure and highlight the urgent need for further research into its hematologic effects in AI/AN populations. Further, these studies are focused on adult populations, and additional investigation in children is warranted. Given cadmium’s known ability to disrupt hematopoietic stem cell function, impair erythropoiesis, and promote leukemogenesis in other populations, targeted surveillance and research in AI/AN communities—particularly among youth—are urgently needed, including samples of blood and urine in children. Community-based participatory research models may be especially well-suited for investigating hematologic impacts in AI/AN populations, ensuring culturally responsive study design and relevance to local health priorities.

## 5. Strategies to Mitigate Consequences of Cadmium Exposure in AI/AN Children

The American Heart Association (AHA) has recognized metal exposure, including cadmium, as a driver of global cardiovascular disease in a Scientific Statement [3]. This Statement also identifies the inequitable exposure to cadmium and the increased risk of metal-associated CVD in minority populations and those of lower socioeconomic status [3]. Another AHA Scientific Statement mentions explicitly the association of cadmium with congenital heart disease, further supporting the importance of addressing environmental toxicants in at-risk populations [63]. An AHA Scientific Statement on the burden of cardiovascular disease and its risk factors in AI/AN adults also highlighted the contributing role of metal exposure [64].

As AI communities have higher cadmium exposure than the general population, it is prudent to address the underlying causes of this issue. Tens of thousands of abandoned hard rock mines on AI lands require decontamination of cadmium [59]. Several technologies have been applied to help with decontamination including phytoremediation with a fast growing and easily cultivated cadmium accumulator plant, seeds of various plants for water purification, washing, leaving, or flushing with chemical agents, adding non-toxic materials to reduce solubility of heavy metals, covering the original pollutants with clean material, and mixing polluted materials with clean materials [17]. Several studies have demonstrated the effectiveness of native plants in the extraction of cadmium in contaminated areas in California and Colorado [65]. The utilization of native plant species has proven cost-effective in phytoremediation, and the inclusion of AI/AN communities in the restoration of traditional landscapes also serves as an effective means of community engagement and leverage of traditional ecological knowledge [66]. To mitigate cadmium exposure in AI/AN communities, policies for federal accountability are paramount.

The United States Environmental Protection Agency (EPA), Federal Drug Administration (FDA), and Occupational Safety and Health Administration (OSHA) have historically worked in tandem to monitor and regulate environmental, workplace, and food-based exposures to cadmium and other metals at the national level [67,68,69]. While congressionally approved and funded, these studies lack enforcement powers and are largely ineffective without executive support.

However, recent actions have limited the enforcement of existing EPA laws and regulations. The Supreme Court’s recent decision to overturn ‘Chevron deference’ significantly limits the ability of federal regulators to interpret and enforce environmental laws without explicit Congressional authorization for each pollutant, potentially delaying enforcement and expanding exposure risks (Loper Bright Enterprises v. Raimondo No. 22-451; Relentless, Inc. v. Department of Commerce, No. 22-1219). While the 2026–2030 EPA Strategic Plan detailing goals to reach this target are not expected to be published until February 2026, the New York Times reported in March 2025 that the EPA planned to dismantle its research arm by firing up to 1155 scientists—nearly 75% of its research staff—in an attempt to reach the goal of a 65% departmental budget cut [70]. Additional exemptions from the Clean Air Act, a landmark law established in 1970 to regulate air pollution and toxic emissions and protect the health of Americans, may also permit energy companies to electively bypass mandated regulations, which may include mining actions and rehabilitation [71].

Limiting the funding required to regulate the impacts of these metals effectively will further undermine agencies’ abilities to protect Americans’ health, as they were initially designed to do. Slashing research personnel and support will not only make it harder to innovate environmental solutions for reduced heavy metal exposure but also it will make it harder to longitudinally monitor heavy metal exposures and health consequences secondary to these new policy changes. In addition, ongoing efforts to mitigate climate change and minimize the increased risk of cadmium infiltration into water systems through flooding and erosion are paramount to limit further contamination.

Representatives at the tribal, state, and federal levels must reaffirm the critical work of these bodies and empower them to safeguard the health of all American citizens, especially AI populations, who are especially proximal and thus vulnerable to the harmful effects of cadmium and other heavy metals on health and development.

## 6. Conclusions

Cadmium exposure remains an underrecognized yet pervasive environmental threat with wide-ranging cardiovascular and hematologic consequences. In children, including those from AI/AN communities, cadmium disrupts normal development through its effects on erythropoiesis, immune regulation, congenital heart development, neurocognitive outcomes, and renal function. These risks are amplified in AI/AN populations, who face disproportionate cadmium exposure due to legacies of environmental injustice, including proximity to contaminated mining sites, limited access to safe foods, and higher rates of tobacco use.

Despite mounting evidence of the toxic potential of cadmium and its relevance for AI/AN children, critical knowledge gaps persist. There is a dearth of pediatric and longitudinal data that link exposure to specific health outcomes in this population. Moreover, the indirect effects of cadmium through renal and epigenetic pathways remain incompletely understood, and virtually no research has specifically examined hematologic disease outcomes in AI/AN children.

Addressing this inequity requires not only further research but also immediate action through policy, environmental remediation, and culturally responsive public health efforts. Smoking cessation programs, decontamination of abandoned mines, dietary safety regulations, and strong tribal sovereignty in environmental decision-making can all be essential components of a multi-pronged strategy to reduce exposure. As climate change continues to exacerbate environmental vulnerabilities, protecting children, especially those in historically marginalized communities, must be a priority for public health and environmental justice.

## Figures and Tables

**Table 1:** Cardiovascular health and cadmium exposure in AI/AN people.

Research Title	Aim/Objective	Methodology	Study Model	Clinical Findings
Cadmium exposure and incident cardiovascular disease [57]	Evaluate the association between urine cadmium levels and cardiovascular disease incidence and mortality in AI cohorts.	Prospective cohort study of 3348 AI adults aged 45–74 years who participated in the Strong Heart Study in 1989–1991. Urine cadmium levels were measured, and the cohort was followed until 31 December 2008; CVD was assessed at follow-up.	Prospective cohort study	The overall median of urine cadmium concentrations at baseline was 0.92, and the geometric mean was 0.94 μg/g creatinine. Relative risk overall for CVD incidence, CHD incidence, stroke incidence, and heart failure incidence was 1.24 (1.11–1.38), 1.22 (1.08–1.38), 1.75 (1.17–2.59), and 1.39 (1.01–1.94), respectively.
Cadmium exposure and incident peripheral arterial disease [58]	Evaluate the association of urine cadmium concentrations with incident peripheral arterial disease in the AI cohort.	Prospective cohort study with 2864 adults AI aged 45–74 years who participated in the Strong Heart Study from 1989 to 1991. Urine cadmium levels were measured, and PAD was defined at follow-up.	Prospective cohort study	The overall geometric mean of urine cadmium concentrations was 0.94 μg/g creatinine. Four hundred seventy cases of incident PAD were identified. Hazard ratio (comparing 80th percentile to 20th percentile of urinary cadmium): 1.41 (1.05–1.81)
Cadmium body burden and increased blood pressure in middle-aged American Indians: the Strong Heart Study [56]	Evaluate the association between urinary cadmium concentrations and blood pressure in AI adults.	Cross-sectional analysis of urinary cadmium concentrations and blood pressure	Prospective cohort study	The overall geometric mean of urine cadmium concentrations was 0.94 μg/g creatinine. There is a “correlation between urinary Cd and smoking pack-year among ever-smokers” (r^2^ = 0.16, *p* < 0.00010). Cross-sectional analysis revealed that urinary in Cd was significantly associated with higher systolic blood pressure. Per 1 unit increase in Cd, there was +1.64 mmHg systolic BP (*p* = 0.002) overall.
Cadmium body burden, hypertension, and changes in blood pressure over time: results from a prospective cohort study in American Indians [18]	Evaluate the association between urinary cadmium levels and their effect on blood pressure in AI communities.	Urine cadmium levels from 3047 Strong Heart Study participants were measured, and corresponding longitudinal changes in blood pressures from 1989 to 1999 were modeled.	Prospective cohort study	A one-unit increase in log(Cd) was correlated with a 10% higher risk of hypertension (95% CI: 1.01–1.20). For the upper quintile of urinary cadmium levels, the estimated change in systolic blood pressure per year was 0.62 mmHg (0.37–0.87) and the estimated change in diastolic blood pressure per year was 0.18 mmHg (0.05–0.31).
